# A Strategy for Controlling Motions Related to Sensory Information in a Walking Robot Big Foot

**DOI:** 10.3390/s23031506

**Published:** 2023-01-29

**Authors:** Ivan Chavdarov, Kaloyan Yovchev, Lyubomira Miteva, Aleksander Stefanov, Dimitar Nedanovski

**Affiliations:** 1 Institute of Robotics, Bulgarian Academy of Sciences, Acad. G. Bonchev St, Bl. 1, 1113 Sofia, Bulgaria; 2 Faculty of Mathematics and Informatics, University of Sofia “St. Kliment Ohridski”, 1504 Sofia, Bulgaria; 3 Institute of Mathematics, Bulgarian Academy of Sciences, Acad. G. Bonchev Str., Bl. 8, 1113 Sofia, Bulgaria; 4 Institute for Nuclear Research and Nuclear Energy, 72 Tsarigradsko Shosse Blvd., 1784 Sofia, Bulgaria

**Keywords:** walking robot, motor control, movement of sensors

## Abstract

Acquiring adequate sensory information and using it to provide motor control are important issues in the process of creating walking robots. The objective of this article is to present control algorithms for the optimization of the walking cycle of an innovative walking robot named “Big Foot”. The construction of the robot is based on minimalist design principles—only two motors are used, with which Big Foot can walk and even overcome obstacles. It is equipped with different types of sensors, with some of them providing information necessary for the realization of an optimized walk cycle. We examine two laws of motion—sinusoidal and polynomial—where we compare the results with constant angular velocity motion. Both proposed laws try to find balance between minimizing shock loads and maximizing walking speed for a given motor power. Experimental results are derived with the help of a 3D-printed working prototype of the robot, with the correct realization of the laws of motion being ensured by the use of a PD controller receiving data from motor encoders and tactile sensors. The experimental results validate the proposed laws of motion and the results can be applied to other walking robots with similar construction.

## 1. Introduction

Mobile robots are designed to function in a complex environment, which requires that they have to possess specific capabilities, such as: climbing stairs [1,2,3]; avoiding obstacles and moving on uneven terrain [4,5]. Many of their applications involve them covering a given area, while bypassing obstacles within it. Some of the tasks that mobile robots are usually designed for include: cleaning [6,7], grass cutting [8], agricultural applications [9], and underwater operations [10]. They are capable of moving in an unstructured and uneven environment [1] and can take part in rescue missions or research inspections. Thus, the use of suitable sensors becomes necessary.

In general, providing adequate sensor input is quite complicated. For example, in [11], an integrated laser-based perception is applied for planning the steps and control of a walking humanoid robot in an unknown rugged terrain. A perception system determines the surrounding environment with an accuracy of several centimeters and the robot’s movements are realized based on input data obtained by scanning with a laser sensor. The authors of [4] consider the issue of perception of an uneven terrain and its mapping with a walking robot equipped with low-cost optical range sensors providing only 2D information. A Hokuyo URG-04LX light sensor and laser scanner are used. The mapping algorithm and methods to remove artifacts that lead to quality errors in the map are applied. Article [12] describes the design and testing of a bipedal robot. Each of its legs is equipped with six servo motors. A gyroscope and an accelerometer are used to measure the current position of the robot’s structure in the space. Control algorithm stabilizes the robot in an upright position. Potentiometers placed in the axes allow measuring of the angular positions of the individual servomechanisms during movement.

An important problem in the control of mobile robots is the preservation of stability. Recovering from a fall is usually hard (often impossible without outside assistance), so measures must be taken to avoid it. Stability is divided into two groups: dynamic and static. Static stability means that the robot maintains balance without constantly making adjustments to its steering. In this case, the projection of the robot’s center of gravity always lies in the support polygon defined by its legs (or wheels). Static stability implies that the robot can stop at any time in its walking cycle without losing balance [13]. To maintain dynamic stability, the robot must actively balance its body. This requires much more complex control algorithms and also usually the robot has a large number of degrees of freedom. The forces and moments in the robot’s legs are an important factor when one investigates dynamical stability [14].

The planning of the gait for walking robots is a complicated task, which needs to be consistent with the terrain [11]. Wayfinding methods proposed in the literature can be divided into two categories: offline and online planning strategies. Offline strategies use a previously known map of the region where the robot performs a certain task, using different path-planning approaches: genetic algorithms [15], cellular decomposition [16], neural networks [17], etc. In the online strategies, obstacles are detected in real time using various sensors [18]. Articles [6,7,8,9,10] consider coverage path planning and obstacle-avoiding algorithms. The goal is to find a path that covers all points in a given region [7]. One of the widely used methods is the Boustrophedon cell decomposition [19]. In [20], the bipedal walking of a robot is realized by a method of control based on information received from various sensors. The control of the walking function uses separation of the movements in the sagittal and lateral planes. The effectiveness of the proposed method is investigated with a walking robot, “BLR-G2”, equipped with pressure sensors in the feet. These sensors provide information about the state of contact with the floor. This contributes to a realization of a smooth walk with a good grip on the surface. Article [21] presents a hexapod robot walking on uneven terrain. A trajectory generator is used for precise control of its legs. Trajectories are further shaped by sensory information. Thus, the robot passes through obstacles of different sizes and rough surfaces. The results show that by integrating the trajectory generator on foot, the sensor-driven six-legged robot can achieve better terrain adaptability and better walking performance. The bipedal robot “Johnny” is designed to achieve a dynamically stable gait, which enables high walking speeds [22]. Very accurate and fast sensors have been developed for this purpose. The design uses six component force-torque sensors. The control scheme is based on the information from these sensors and the robot can walk on an unstructured terrain.

Compared with the wheeled robots, the walking robots have much more complex structure, have more motors, and are slower. However, they are able to overcome higher and more complicated obstacles. There are studies that aim to reduce the complexity of the walking robots while maintaining their advantages. The authors of [23] present a conceptual design of a new minimalist biped walking robot with four degrees of freedom. The proposed mechanism combines sensing the stability and balancing of the robot during the steps. The sensor mechanism uses an additional flexible ankle joint that is able to provide a measurement of the instability of the biped robot. A balance mass and control algorithm are used to maintain the lateral stability of the robot. The authors of [24] propose a concept for a bipedal robot with vertical stabilization of the robot base and minimization of its lateral oscillations. This robot uses only six actuators and has a good energy balance compared with purely articulated biped robots.

The above literature review for the walking robots could be summarized as follows:

- Since their primary function is to work in an undefined and complex environment, they have to perform complicated spatial movements, which usually requires a sophisticated mechanical design;
- In order to obtain appropriate information for the environment, they have to be equipped with a sufficient number of different types of sensors;
- The control system has to be able to handle the processing of a large amount of sensory information including the motion planning algorithms and changing conditions of the unstructured environment.

Mechanical designs of robots with a large number of degrees of freedom, sensors, and complex control algorithms are often used to solve these problems. However, such an approach leads to significant disadvantages: low reliability, need for more energy, and high production and maintenance cost.

We ask the following questions:

- Could we use the robot’s movements in order to obtain more information from its sensors?
- What is the minimal number of degrees of freedom which allows for a creation of a walking robot with good functional capabilities?
- In which way can sensory information be used to improve the walking performance of the robot?

We propose an innovative design with only two degrees of freedom called “Big Foot”. For this design, the first question is examined in article [25]. The answers to the remaining questions depend on details that must be further specified, i.e., what is the expected walking environment, expected capabilities of the robot, cost, etc. Our proposed design is capable of walking on even or uneven surfaces using only two motors and having a low overall cost. The aim of this paper is to try to optimize the motion of the robot for the case of walking on a plane (or a surface that is close to a plane) by examining two types of laws of motion: polynomial and sinusoidal. The overall goal is to find a compromise that achieves sufficient walking speed, while keeping impact shocks low, and is compatible with motor power constraints. The results are verified experimentally by using a 3D-printed prototype. The realization of the desired laws of motion is ensured by the use of a PD controller reading data from the motor encoders and the tactile sensors on the robot’s feet.

This paper is structured as follows: Section 2 examines the overall structure of the robot and some of the previous work on the subject; Section 3 presents in detail the structure of the walking mechanism and its kinematics. The used laws of motion are also located here. This section also contains the experimental setup and details on the 3D-printed prototype and motors and sensors used; Section 4 contains the experimental results and their comparison with the theoretical laws of motion; Section 5 is a short discussion; Section 6 contains some concluding remarks; and Section 7 provides the patents, Supplementary Materials, and other information.

## 2. Background and Related Work

**Figure 1:**
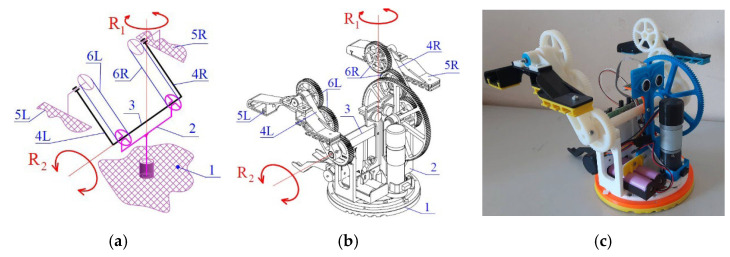
The robot Big Foot: principle scheme (**a**); 3D model (**b**); photo of a 3D-printed prototype (**c**).

The development of the robot in question went through a few iterations, with the original idea first presented in articles [26,27] and patent [28]. The robot has only two degrees of freedom. It consists of a base (base 1), on which the body (body 2) of the robot is connected by means of a vertical rotary joint (with axis R1), in which a shaft (shaft 3) is mounted, which drives the symmetrical arms (legs) 4R and 4L of the robot. Shaft 3 is perpendicular to axis R1. In Figure 1a, the principle scheme of the robot is given; 1b is a 3D model; and 1c is a photo of a 3D-printed robot prototype.

In the two symmetrical arms (legs) 4L and 4R, the robot feet 5L and 5R are bearing mounted. The rotations R1 and R2 are driven by DC motors and transmission mechanisms. The parts identified as 6R and 6L are two belts or gears that provide parallel movement of the feet 5L and 5R relative to base 1.

Although the robot has only two motors and a relatively simple design, it moves by walking and rotates on 360[°], avoids obstacles, and even climbs stairs suitable to its size. In [25], the main kinematic dependencies of the robot are presented and a design based on a proportional distribution of the potential energy during the movement of the robot is proposed. A simulation of its movements while climbing an obstacle is given. The robot’s ability to passively adapt to high obstacles in order to overcome them is discussed. The authors of [29] present numerous experiments with 3D-printed models of the robot with different shapes and materials of the feet, which lead to an increase in its movement capabilities in a complicated environment. The dynamics of the robot is developed in [30].

**Figure 2:**
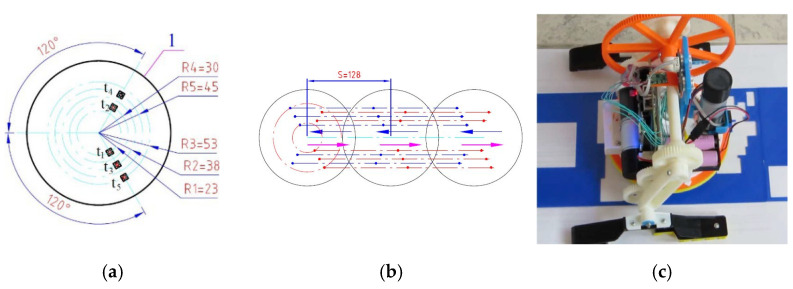
Location of the tactile sensors ($t_{i};\;i=1-5$) on the base of the robot “Big Foot” (**a**); exemplary trajectory for scanning of bumps (**b**); experiment using the robot for scanning light unevenness on the surface (**c**).

Although the mechanics of the robot is relatively well studied both theoretically and experimentally, its sensor systems and possibilities for exploring the surrounding environment are discussed in only one article [25]. The 3D printed model of the “Big Foot” robot has five tactile sensors and one force sensor attached to the bottom of the circular base 1 (see Figure 2a). When the robot moves, it steps on the sensors and activates the tactile buttons. As there may be bumps on the surface the robot is moving on, one or more of the buttons may not be pressed and activated. Thus, the walking robot can be used to detect surface irregularities or to “read” inscriptions or drawings that are embossed or indented in the surface (Figure 2b,c).

The location of the tactile sensors is chosen in such a way that the robot could measure up to five different points on the surface each time it touches it. The skillful combination of the two rotational movements of the robot (R1 and R1) with the sensors at its base are used to enrich the received sensory information. In [25], such a strategy for the study of irregularities with tactile sensors is considered, and a video with the programmed movements can be seen in the following link: https://youtu.be/RYRJZcYdIX0 (accessed on 20 January 2023).

The robot is also equipped with other sensors: magnetic encoders of the motors, a gyroscope, a magnetometer, and an accelerometer located in body 2.

Here, we further develop the idea presented in [25] to show that sensory signals combined with the movements of a robot based on a minimalist design are useful both for receiving external information and for control of the robot’s walking movements.

A word on notations: we will use degrees for angles where possible (for example in graphics) but will switch to radians when needed.

## 3. Materials and Methods

The main methods, which we apply to develop and test the strategy for managing the information received from the walking robot’s sensors are as follows: application of kinematics to determine the necessary motions, velocities, and laws for motor control; mathematical analysis for defining a suitable time dependence of the velocity, which ensures smooth robot movement; synthesis of the control algorithm based on sensor information; design, 3D modeling, and printing of a prototype for experiments; and experimental validation.

### 3.1. Kinematics of Walking

**Figure 3:**
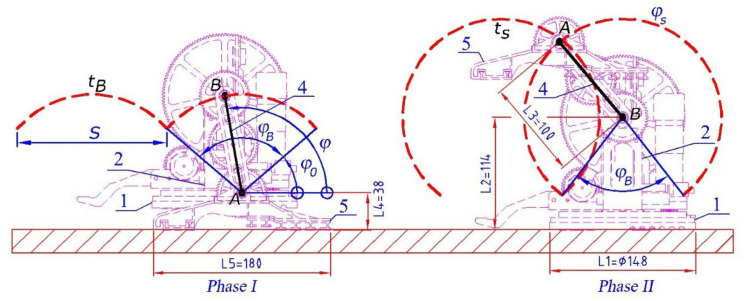
Walking phases (on even horizontal surface) for the robot “Big Foot”. The trajectory of the base (robot’s body) is $t_{B}$ while *t*_*s*_ is the trajectory of the robot’s feet. The dimensions *L_j_* = 1 ÷ 5, are in millimeters.

In [27], we consider the kinematics of our walking robot. There are two phases of the walking function (see Table 1). The walking mechanism occurs only in the motor which turns shaft 3 (Figure 1). For one revolution of shaft 3, body 1 of the robot is successively moved along an arc corresponding to the angle $\varphi_{B}$ and feet 5 along the arc $\varphi_{S}$ (Figure 3). These angles are defined as a function of the step length S of the robot:

(1) $$\varphi_{B}=2arctan\left(\frac{S}{2\left(L_{2}-L_{4}\right)}\right),$$

(2) $$\varphi_{S}=2\pi -\varphi_{B}.$$

$L_{2}$ and $L_{4}$ are the distances shown in Figure 3 and step S is defined as [26]:

(3) $$s=2\sqrt{L_{3}^{2}-{\left(L_{2}-L_{4}\right)}^{2}}$$

As a result of Equations (1)–(3), for both phases of walking, the angles $\varphi_{B}$ and $\varphi_{S}$ (rotation angle of link 2) depend only on the dimensions $L_{2},\;L_{3}$, and $L_{4}$ of the links 1, 2, 4, and 5.

The movements of the robot are carried out successively as follows. In the first phase of walking the robot rests on feet 5 and in the second phase it rests on base 1. During the transition between the first and second phase, the instantaneous center of velocity of arm 4 changes with a jump from point B’s instantaneous center of velocity to point A. Thus, the elements of the robot undergo shock loads during this transition.

If we assume that the motor maintains a constant speed of shaft 3, which drives link 4, then for the robot’s velocity $v_{x}$ on a horizontal plane, we have:

(4) $$v_{x}={\dot{x}}_{c1}=\omega L_{3}\sin\left(\varphi \right).$$

**Figure 4:**
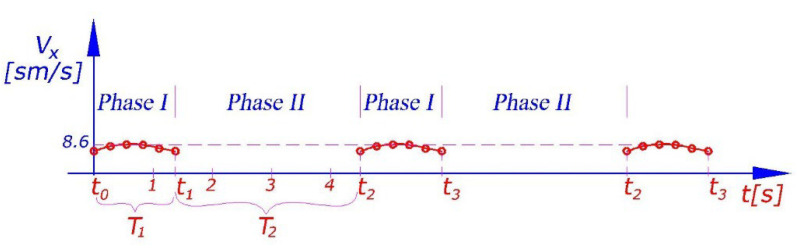
Change in the robot’s linear velocity $v_{x}$ over time obtained according to Figure 3 and Equation (4) in the case of the uniform rotation of shaft 3 with the angular velocity ω=50 [deg/s].

Note that there is forward movement only during the first phase of walking (when the feet are on the ground). During this phase, we have $\varphi =\omega t+\varphi_{0}$. The robot’s velocity–time graph is provided in Figure 4.

The robot’s velocity $v_{x}$ is a periodic function of time with the period $T=T_{1}+T_{2}=t_{2}-t_{0}$ (see Figure 4). This period is divided into two parts. In the first part, $T_{1}=t_{1}-t_{0}$, the robot’s feet 5 are on the ground and the robot is moving. In the second part, $T_{2}=t_{2}-t_{1}$, the robot’s base/body (links 1 and 2) are on the ground and arm 4 and feet 5 are rotating (see Figure 3). Obviously the time, $t_{2}-t_{1}$, in which feet do not touch the ground should be minimized. A generalized overview of the robot’s movement is provided in Table 1.

### 3.2. Law of Motion Synthesis

In order to find an appropriate control law for the motor, which drives the walking mechanism, we are guided by the following ideas/aims: the robot’s movement should be as fast as possible; impact loads in the transition between the two walking phases must be minimal; and the movement should be smooth and the available sensors should be used in the control process. The shock phenomenon is observed when a sudden (instantaneous) change in the speed of a body is caused by the action of instantaneous forces. The impact force reaches large magnitudes during the collision process. The momentum of the impact leads to a step change in the velocity of the body [31]:

(5) $$J=m\left(v-v_{0}\right)=\underset{\tau \to 0}{\lim}\overset{t_{0}+\tau }{\underset{t_{0}}{\int }}Fd\tau ,$$

where J is the impulse of the force F, v is the velocity at a moment of time $t_{0}+\tau$, which is very close to the moment of time $t_{0}$ at which the contact between the two bodies occurs, *m* is the mass of the body, and τ is a short interval of time. In our case, at the moment of contact, $v_{0}=0$, since the body becomes immobile. If we reduce the velocity v at the time $t_{0}+\tau$, which is very close to the contact time, we will reduce the impact load. Moreover, if the velocity changes smoothly, we will have small inertial forces. For these reasons, we require the following initial conditions for the angle $\varphi_{1}$, the angular velocity ${\dot{\varphi }}_{1}$, and the angular acceleration ${\ddot{\varphi }}_{1}$ (the dots denote the time derivative) for phase I (feet are on the ground):

(6) $$\varphi_{1}\left(t_{0}\right)=\varphi_{1}\left(0\right)=\varphi_{0},\;\;\;\varphi_{1}\left(t_{1}\right)=\varphi_{1}\left(T_{1}\right)=\varphi_{B}+\varphi_{0},$$

(7) $${\dot{\varphi }}_{1}\left(t_{0}\right)={\dot{\varphi }}_{1}\left(0\right)=0,\;\;\;{\dot{\varphi }}_{1}\left(t_{1}\right)={\dot{\varphi }}_{1}\left(T_{1}\right)=0,$$

(8) $${\ddot{\varphi }}_{1}\left(t_{0}\right)={\ddot{\varphi }}_{1}\left(0\right)=0,\;\;\;{\ddot{\varphi }}_{1}\left(t_{1}\right)={\ddot{\varphi }}_{1}\left(T_{1}\right)=0.$$

Figure 3 and Figure 4 explain the parameters in Equations (6)–(11). In a similar way, for the phase II robot’s base on the ground we have:

(9) $$\varphi_{2}\left(t_{1}\right)=\varphi_{2}\left(0\right)=\varphi_{B}+\varphi_{0},\;\;\;\varphi_{2}\left(t_{2}\right)=\varphi_{2}\left(T_{1}+T_{2}\right)=\varphi_{B}+\varphi_{S}+\varphi_{0},$$

(10) $${\dot{\varphi }}_{2}\left(t_{1}\right)=0,\;\;\;{\dot{\varphi }}_{2}\left(t_{2}\right)={\dot{\varphi }}_{2}\left(T_{1}+T_{2}\right)=0,$$

(11) $${\ddot{\varphi }}_{2}\left(t_{1}\right)=0,\;\;\;{\ddot{\varphi }}_{2}\left(t_{2}\right)={\ddot{\varphi }}_{2}\left(T_{1}+T_{2}\right)=0$$

The motor’s limitations and load should also be taken into account. During each phase, the motor can achieve angular accelerations in the interval $0\leq \left|\dot{\varphi }\left(t\right)\right|\leq \omega_{max}$ and angular accelerations in the interval $0\leq \left|\ddot{\varphi }\left(t\right)\right|\leq \varepsilon_{max}$.The maximal values are determined by the power of the motor and the moments of inertia of the corresponding links.

We consider two types of time dependence for the angular velocity which meet the conditions stated above: sinusoidal and polynomial.

#### 3.2.1. Sinusoidal Dependence

The trigonometric functions sine and cosine are suitable for constructing a law of motion, which smoothly varies the velocity from zero to maximum and then decreases it again to zero. A smooth increase in the angular velocity ω when starting from rest and a smooth stop can be ensured if we use a function of the form:

(12) $$\omega \left(t\right)=\dot{\varphi }\left(t\right)=A-Acos\left(kt\right).$$

Here, A is the amplitude of the angular velocity and k defines the frequency. For the separate phases I and II of motion we are only interested in one period of the function in Equation (12). After integration, for the law of motion of link (arm) 4 we obtain:

(13) $$\varphi \left(t\right)=At-A\frac{1}{k}sin\left(kt\right)+C.$$

The constant C sets the initial angle of rotation for the phases I and II. For the angular acceleration ε of link 4 we have:

(14) $$\varepsilon \left(t\right)=\ddot{\varphi }\left(t\right)=Aksin\left(kt\right).$$

During phase I time is in the interval $t\in \left[0,T_{1}\right]$. The coefficient k is determined from Equations (7) and (12), $k=\frac{2\pi }{T_{1}}$. The constant C is determined by the first condition in Equation (6). We obtain $C=\varphi_{0}$ if the robot’s base 1 is on the ground and the motor rotates the links (4L and 4R). The angle $\varphi_{0}$ corresponds to the moment when the phase of movement changes, which is read by the tactile sensors (see Figure 3). From the second condition in Equations (6) and (13) we obtain: $A=\frac{\varphi_{B}}{T_{1}}$. Thus, we could write Equations (12)–(14) for phase I in the form:

(15) $$\varphi_{1}\left(t\right)=\frac{\varphi_{B}}{T_{1}}\left[t-\frac{T_{1}}{2\pi }sin\left(\frac{2\pi }{T_{1}}t\right)\right]+\varphi_{0},$$

(16) $${\dot{\varphi }}_{1}\left(t\right)=\frac{\varphi_{B}}{T_{1}}\left[1-cos\left(\frac{2\pi }{T_{1}}t\right)\right],$$

(17) $${\ddot{\varphi }}_{1}\left(t\right)=\frac{2\pi \varphi_{B}}{T_{1}^{2}}sin\left(\frac{2\pi }{T_{1}}t\right).$$

During this phase, the maximal angular velocity is ${\dot{\varphi }}_{1max}=2\frac{\varphi_{B}}{T_{1}}$ and is reached at time $t=\frac{T_{1}}{2}$. The maximal angular acceleration ${\ddot{\varphi }}_{1max}=\frac{\varphi_{B}}{T_{1}^{2}}2\pi$ is reached at *t* = $\frac{T_{1}}{4}$, and with the opposite sign at $t=\frac{3T_{1}}{4}$. If the maximal angular velocity and acceleration are known, then one could determine the least possible time, $T_{1min}$, for the execution of phase I:

(18) $$T_{1min}=\min\left[\begin{array}{cc} \frac{2\varphi_{B}}{{\dot{\varphi }}_{1max}}, & \sqrt{\frac{2\pi \varphi_{B}}{{\ddot{\varphi }}_{1max}}} \end{array}\right].\;$$

Since the angle $\varphi_{B}$ is significantly smaller than $\varphi_{S}$ and $\varphi_{B}+\varphi_{S}=2\pi$, usually $T_{1min}=\left|\sqrt{\frac{2\pi \varphi_{B}}{{\overset{´}{\varphi }}_{1max}}}\right|$.

In a similar way, using Equations (9)–(14) for phase II, corresponding to time $t\in \left[T_{1},T_{1}+T_{2}\right]$, we obtain: $k=\frac{2\pi }{T_{2}}$, $C=\varphi_{0}+\varphi_{B}$‚ $A=\frac{\varphi_{s}}{T_{2}}$. Reaching the angle $\varphi_{0}+\varphi_{B}$ is confirmed by the tactile sensors in the robot’s base 1. Equations (12)–(14) for phase II are modified as follows:

(19) $$\varphi_{2}\left(t\right)=\frac{\varphi_{S}}{T_{2}}\left[\left(t-T_{1}\right)-\frac{T_{2}}{2\pi }sin\left(\frac{2\pi }{T_{2}}(t-T_{1}\right)\right]+\varphi_{0}+\varphi_{B},$$

(20) $${\dot{\varphi }}_{2}\left(t\right)=\frac{\varphi_{S}}{T_{2}}\left[1-cos\left(\frac{2\pi }{T_{2}}(t-T_{1}\right)\right],$$

(21) $${\ddot{\varphi }}_{2}\left(t\right)=\frac{2\pi \varphi_{S}}{T_{2}^{2}}sin\left(\frac{2\pi }{T_{2}}(t-T_{1}\right).$$

Here, the maximal angular velocity is ${\dot{\varphi }}_{2max}=2\frac{\varphi_{S}}{T_{2}}$ and is reached at time $t=T_{1}+\frac{T_{2}}{2}$. The maximal angular acceleration, ${\ddot{\varphi }}_{2max}=\frac{\varphi_{S}}{T_{2}^{2}}2\pi$, is reached at *t* =$\;T_{1}+$ $\frac{T_{2}}{4}$, and with the opposite sign at и $t=T_{1}+\frac{3T_{2}}{4}$. Again, if the maximal angular velocity and acceleration are known, then one could determine the least possible time, $T_{2min}$, for the execution of phase II:

(22) $$T_{2min}=\min\left[\begin{array}{cc} \frac{2\varphi_{S}}{{\dot{\varphi }}_{2max}}, & \sqrt{\frac{2\pi \varphi_{S}}{{\ddot{\varphi }}_{2max}}} \end{array}\right].\;$$

Since $\varphi_{S}>\varphi_{B}$, this phase is performed in a longer time compared with phase I and it is expected that the motor will reach its maximal angular velocity, which corresponds to $T_{2min}=\frac{2\varphi_{S}}{{\dot{\varphi }}_{2max}}$.

#### 3.2.2. Polynomial Dependence

Another suitable function for a smooth variation in the angular velocity ω during the change in the phases of movement is a polynomial of degree four:

(23) $$\omega \left(t\right)=\dot{\varphi }\left(t\right)=a_{1}t^{4}+a_{2}t^{3}+a_{3}t^{2}+a_{4}t+a_{5}.$$

Such a polynomial has at most 3 extreme points. The analysis is similar to that of the sinusoidal law. After integration, we obtain for the law of motion:

(24) $$\varphi \left(t\right)=\frac{a_{1}t^{5}}{5}+\frac{a_{2}t^{4}}{4}+\frac{a_{3}t^{3}}{3}+a_{5}t+C,$$

Here, C is a constant of integration which again sets the initial angle of rotation for the phases I and II. After differentiating (23), for the angular acceleration ε of link 4 we have:

(25) $$\varepsilon \left(t\right)=\ddot{\varphi }\left(t\right)=4a_{1}t^{3}+3a_{2}t^{2}+2a_{3}t+a_{4},$$

We determine the coefficients $a_{i}$ and the constants of integration from Equations (6–11). Again, we will review each phase separately, and reaching the angle $\varphi_{0}$ corresponds to the moment when the phases of movement change.

During phase I time is in the interval $t\in \left[0,T_{1}\right]$. We have 6 coefficients $a_{1},\ldots ,\;a_{5},\;C$ and 6 Equations (6)–(8), and solving them leads to:

(26) $$\varphi_{1}\left(t\right)=\varphi_{B}\left(\frac{10t^{3}}{T_{1}{}^{3}}+\frac{-15t^{4}}{T_{1}{}^{4}}+\frac{6t^{5}}{T_{1}{}^{5}}\right)+\varphi_{0},$$

(27) $${\dot{\varphi }}_{1}\left(t\right)=\frac{\varphi_{B}}{T_{1}}\left(\frac{30t^{2}}{T_{1}{}^{2}}+\frac{-60t^{3}}{T_{1}{}^{3}}+\frac{30t^{4}}{T_{1}{}^{4}}\right),$$

(28) $${\ddot{\varphi }}_{1}\left(0\right)=\frac{\varphi_{B}}{T_{1}{}^{2}}\left(\frac{60t^{\;}}{T_{1}{}^{\;}}+\frac{-180t^{2}}{T_{1}{}^{2}}+\frac{120t^{3}}{T_{1}{}^{3}}\right).$$

During this phase, the maximal angular velocity is ${\dot{\varphi }}_{1max}=\frac{15}{8}\frac{\varphi_{B}}{T_{1}}$ and is reached at time $t=\frac{T_{1}}{2}$. The maximal angular acceleration, ${\ddot{\varphi }}_{1max}=\frac{10}{\sqrt{3}}\frac{\varphi_{B}}{T_{1}^{2}}$, is reached at $t=\left(\frac{1}{2}-\frac{\sqrt{3}}{6}\right)T_{1}$, and with the opposite sign at $t=\left(\frac{1}{2}+\frac{\sqrt{3}}{6}\right)T_{1}$. Again, if the maximal angular velocity and acceleration are known, then one could determine the least possible time, $T_{1min}$, for the execution of phase I:

(29) $$T_{1min}=\min\left[\begin{array}{cc} \frac{15}{8}\frac{\varphi_{B}}{{\dot{\varphi }}_{1max}}, & \sqrt{\frac{10}{\sqrt{3}}\frac{\varphi_{B}}{{\ddot{\varphi }}_{1max}}} \end{array}\right]\;$$

This was the case for the sinusoidal time dependence since the angle $\varphi_{B}$ is significantly smaller than $\varphi_{S}$ and $\varphi_{B}+\varphi_{S}=2\pi$, usually $T_{1min}=\sqrt{\frac{10}{\sqrt{3}}\frac{\varphi_{B}}{{\ddot{\varphi }}_{1max}}}$.

In a similar way, using Equations (9)–(11) and (23)–(25) for phase II, corresponding to time $t\in \left[T_{1},T_{1}+T_{2}\right]$, we obtain:

(30) $$\varphi_{2}\left(t\right)=\varphi_{S}\left(\frac{10{\left(t-T_{1}\right)}^{3}}{T_{2}{}^{3}}+\frac{-15{\left(t-T_{1}\right)}^{4}}{T_{2}{}^{4}}+\frac{6{\left(t-T_{1}\right)}^{5}}{T_{2}{}^{5}}\right)+\varphi_{0}+\varphi_{B},$$

(31) $${\dot{\varphi }}_{2}\left(t\right)=\frac{\varphi_{S}}{T_{2}}\left(\frac{30{\left(t-T_{1}\right)}^{2}}{T_{2}{}^{2}}+\frac{-60{\left(t-T_{1}\right)}^{3}}{T_{2}{}^{3}}+\frac{30{\left(t-T_{1}\right)}^{4}}{T_{2}{}^{4}}\right),$$

(32) $${\ddot{\varphi }}_{2}\left(0\right)=\frac{\varphi_{S}}{T_{2}{}^{2}}\left(\frac{60{\left(t-T_{1}\right)}^{\;}}{T_{2}{}^{\;}}+\frac{-180{\left(t-T_{1}\right)}^{2}}{T_{2}{}^{2}}+\frac{120{\left(t-T_{1}\right)}^{3}}{T_{2}{}^{3}}\right).$$

The maximal angular velocity is ${\dot{\varphi }}_{2max}=\frac{15}{8}\frac{\varphi_{S}}{T_{2}}$ and is reached at time $t=T_{1}+\frac{T_{2}}{2}$. The maximal angular acceleration, ${\ddot{\varphi }}_{2max}=\frac{10}{\sqrt{3}}\frac{\varphi_{B}}{T_{2}^{2}}$, is reached at $t=T_{1}+\left(\frac{1}{2}-\frac{\sqrt{3}}{6}\right)T_{2}$, and with the opposite sign at $t=T_{1}+\left(\frac{1}{2}+\frac{\sqrt{3}}{6}\right)T_{2}$. For the minimal time for execution we have:

(33) $$T_{2min}=\min\left[\begin{array}{cc} \frac{15}{8}\frac{\varphi_{B}}{{\dot{\varphi }}_{2max}}, & \sqrt{\frac{10}{\sqrt{3}}\frac{\varphi_{B}}{{\ddot{\varphi }}_{2max}}} \end{array}\right].\;$$

As in the sinusoidal case, this phase is performed in a longer time compared with phase I and it is expected that the motor will reach its maximal angular velocity, which corresponds to $T_{2min}=\frac{15}{8}\frac{\varphi_{B}}{{\dot{\varphi }}_{2max}}$.

#### 3.2.3. Experiment

**Figure 5:**
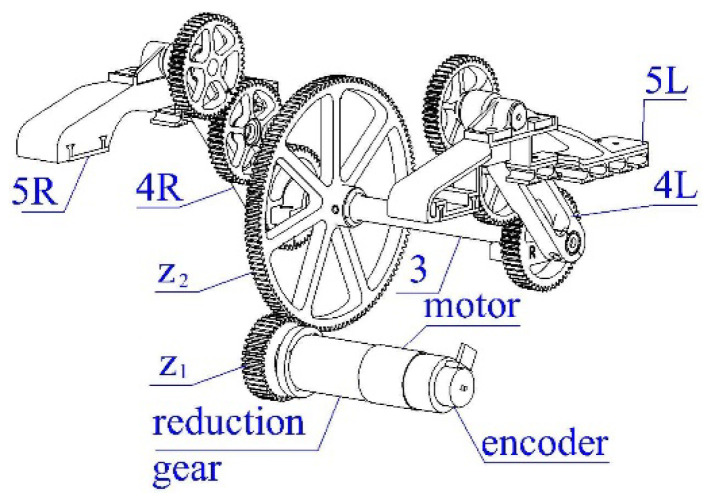
Walking mechanism.

The considered construction of the “Big foot” robot uses two motor reducers of type FIT0277 (12 V-Motor: DC; with encoder, with gearbox; 12VDC; 230 mA; 146 rpm; 51:1) with magnet encoders. The output shaft revolutions are 146 RPM. From the transmission’s (see Figure 5) gear ratio, $i=\frac{z_{2}}{z_{1}}=\frac{124}{40}=3.1$, of the motor’s parameters, we obtain the maximal value for the angular velocity $\omega_{max}={\dot{\varphi }}_{max}=150\left[{}^{\circ}/s\right]$ and angular acceleration $\varepsilon_{max}={\ddot{\varphi }}_{max}=130\left[{}^{\circ}/s^{2}\right]$ for the angular acceleration of the links 4L and 4R. Thus, we are able to determine the least possible durations, $T_{1min},T_{2min}$, for the phases I and II.

Angular velocity control is realized by feedback with a PD-type controller. This controller receives as input the error between the set angular velocity and the current angular velocity, measured in number of encoder readings. The output of the PD controller is the necessary correction of the signal supplied to the motor driver. The motor driver receives as input an integer from 0 to 255. The controller parameters are experimentally set to P = 0.05 и D = 0.00025. P is the proportional term, D is the derivative gain. The proportional term produces an output value that is proportional to the current error value. The derivative of the process error is calculated by determining the slope of the error over time and multiplying this rate of change by the derivative gain. In the transition between the two stages of the movement, the shock load on the robot structure is maximal and there is the greatest need for correction of the input value to the motor driver.

The robot is equipped with tactile sensors in the base (Figure 1a), which allow the moment of contact of the base with the surface to be accurately registered and to determine the phase of the movement. A sensor for measuring acceleration (accelerometer) is also installed on the robot. This sensor allows us to read the acceleration along the vertical *z* axis that acts on the structure when the transition between the two phases takes place. The sensor is set to read values between ±2 g, where $g=9.81\left[m/s^{2}\right]$ is the gravitational acceleration. When the robot is at rest, the sensor reads that the gravitational acceleration and its readings are equal to 1 g, respectively.

Two types of experiments were conducted. In the first type, the constant angular velocity of the motor is set, in which arm 4 of the robot has the angular velocities: $\omega_{1}=118\left[{}^{\circ}/s\right]$ and $\omega_{2}=59\left[{}^{\circ}/s\right]$.

The second type are the experiments with angular velocity control according to Equations (15)–(17) and (19)–(21), subject to restrictions (6–11) and the maximal allowed angular velocity and acceleration for link 4.

## 4. Results

From Equation (3) for the robot’s step we obtain $S=128\left[mm\right]$ and the rotation range of the base in phase I is determined by Equation (2). Thus, the maximal angles are $\varphi_{B}=80.5\left[{}^{\circ}\right]\;$ and $\varphi_{S}=279.5\left[{}^{\circ}\right]$. These are results calculated theoretically using the designed dimensions of the robot. In order to specify these parameters, measurements have been made based on the information from the motor’s encoder. Experimentally, we have found that the encoder takes 4575 readings per full revolution of $360\left[{}^{\circ}\right]$ of arm 4 and feet 5. Thus, one encoder reading equals 0.0787 degrees. Maximal angular velocity (when the feet are in the air) of 1650 readings per second has been experimentally confirmed, which corresponds to $130\left[{}^{\circ}/s\right]$. The angle $\varphi_{B}$ of phase I (when the feet are on the ground and the base is in the air) is 975 encoder readings, i.e., $\varphi_{B}=77\left[{}^{\circ}\right]$. The angle $\varphi_{S}$ of phase II is 3600 encoder readings, i.e., $\varphi_{s}=283\left[{}^{\circ}\right]$.

Equations (18), (22), (29), and (33) determine the times $T_{1min}$, $T_{2min}$, and the periods in Equations (12) and (23). The results are presented in Table 2.

**Figure 6:**
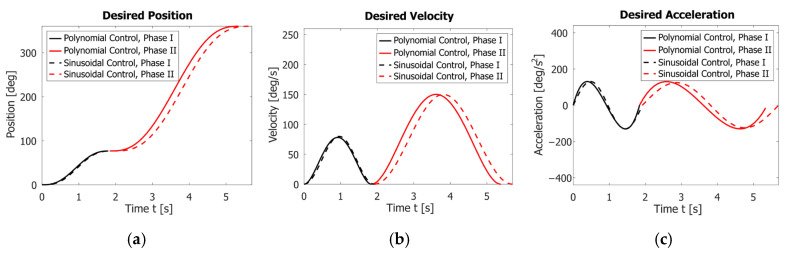
Comparison of the assignment angle positions φ for the two control laws (**a**). Comparison of the assigned angle velocities $\dot{\varphi }$ (**b**). Comparison of the assigned angle accelerations $\ddot{\varphi }$ (**c**).

Thus, Equations (15)–(22) set the sinusoidal motor control laws, while Equations (26)–(33) set the polynomial motor control laws. Figure 6a–c shows a comparison of the angular position, velocity, and acceleration assignments during the entire motion for one period, T, under the two control laws.

**Figure 7:**
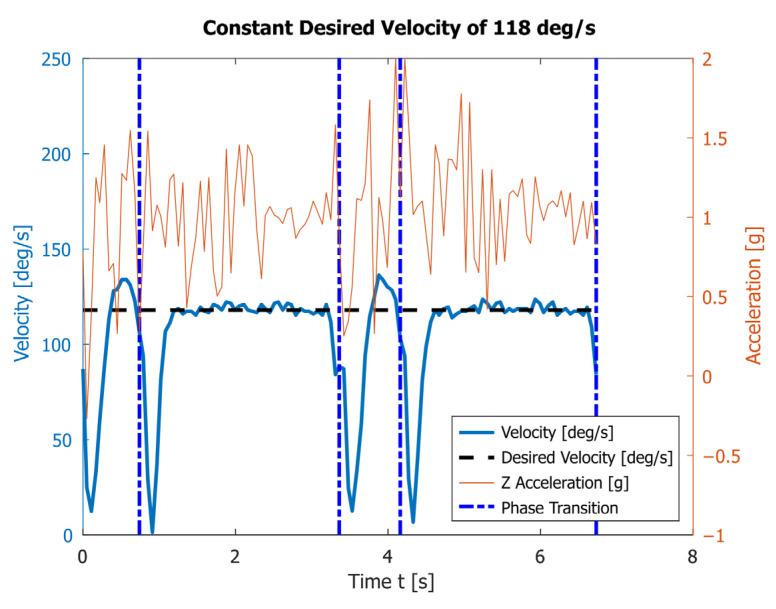
The angular velocity read by the encoder and the vertical acceleration obtained by the accelerometer during the motion of link 4 with a constant angular velocity y $\;\omega_{1}=118\left[{}^{\circ}/s\right]$.

The following figures present raw unfiltered data from the accelerometer and encoder. Figure 7 contains the results of performing two rotations of the robot’s feet at a set constant angular velocity $\omega_{1}=118\left[{}^{\circ}/s\right]$, which is close to the maximal one. At this rate, the average execution times for phases I and II are $T_{1}=0.80\left[s\right]$ and $T_{2}=2.56\left[s\right]$, respectively. It takes an average of $T=3.36\left[s\right]$ for a full walk cycle. During the movement, the robot experiences the following minimal and maximal acceleration values along the *z* axis (the axis normal to the walking plane): −0.26 g and 1.99 g, reported by the accelerometer (Figure 7). These values subject the robot to a strong external load and are not suitable when it is used for a long time.

**Figure 8:**
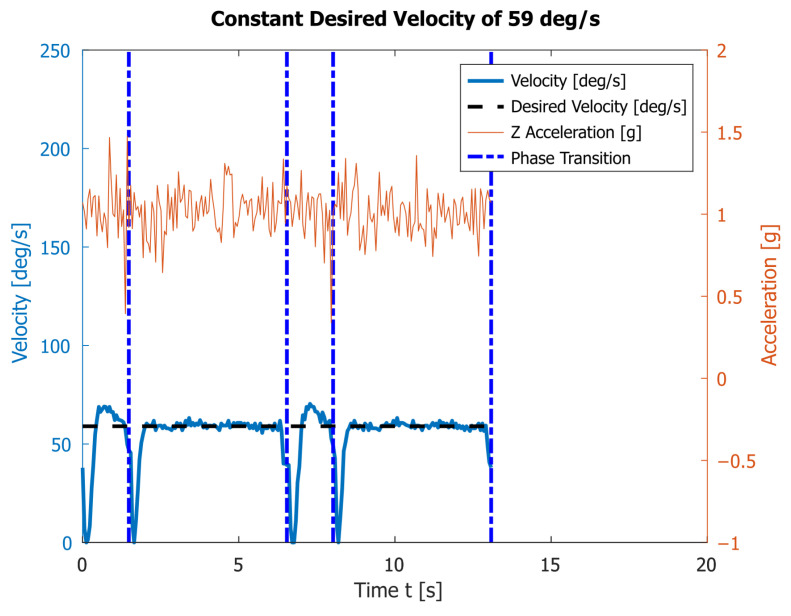
The angular velocity read by the encoder and the vertical acceleration obtained by the accelerometer during the motion of link 4 with a constant angular velocity $\omega_{2}=59\left[{}^{\circ}/s\right]$.

Next, we reduced the constant angular velocity by half to $\omega_{1}=59\left[{}^{\circ}/s\right]$. Figure 8 presents the results of two complete rotations of the robot’s legs. The average execution times of phases I and II are respectively $T_{1}=2.00\left[s\right]$ and $T_{2}=5.06\left[s\right]$. It takes an average of $T=7.06\left[s\right]$ for one full walk cycle. During the movement, the robot experiences the following minimal and maximal acceleration along the *z* axis: 0.34 g and 1.47 g.

**Figure 9:**
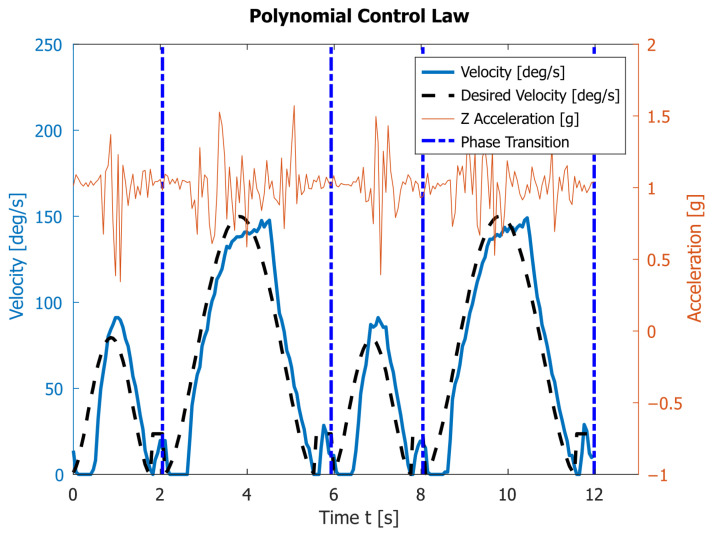
The angular velocity read by the encoder and the vertical acceleration obtained by the accelerometer during the motion of link 4 subjected to the polynomial control law.

The third experiment uses the polynomial control law. Now velocities are set in a way ensuring that at the start and at the end of both phase I and phase II the velocities and accelerations are zero. The results of two complete rotations of the robot’s legs are shown in Figure 9. With the motion planned in this way, the average execution times of phases I and II are $T_{1}=2.11\left[s\right]\;$ and $T_{2}=3.88\left[s\right]$, respectively. It takes an average of $T=5.99\left[s\right]$ for one full walk cycle. During the movement, the robot experiences the following minimal and maximal accelerations along the *z* axis: 0.34 g and 1.57 g. The achieved maximal angular velocity is $\omega_{max}=149\left[{}^{\circ}/s\right]$.

**Figure 10:**
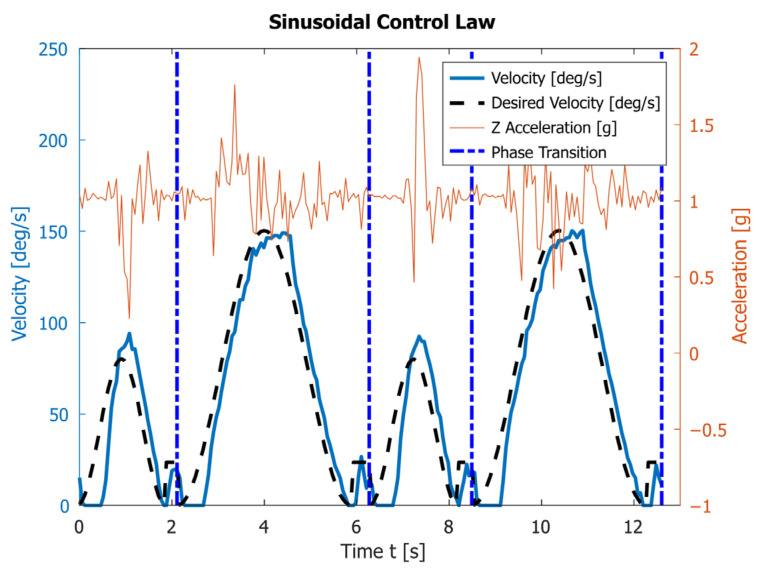
The angular velocity read by the encoder and the vertical acceleration obtained by the accelerometer during the motion of link 4 subjected to sinusoidal control law.

The fourth experiment uses the sinusoidal control law. Again, the velocities are set in a way ensuring that at the start and at the end of both phase I and phase II the velocities and accelerations are zero. The results of two complete rotations of the robot’s legs are shown in Figure 10. The average execution times of phases I and II are $T_{1}=2.17\left[s\right]$ and $T_{1}=4.16\left[s\right]$, respectively. It takes an average of $T=6.32\left[s\right]$ for one full rotation. During the movement, the robot experiences the following minimal and maximal accelerations along the *z* axis: 0.22 g and 1.94 g. The achieved maximal angular velocity is $\omega_{max}=150\left[{}^{\circ}/s\right]$.

In the third and fourth experiments, the robot experiences lower acceleration along the *z* axis during the entire motion compared with the motion at velocity $\omega_{1}=118\left[{}^{\circ}/s\right]$. In real conditions, as seen in Figure 9 and Figure 10, the robot needs a minimal additional time of about 0.30 s on average for transition between the two phases. This is due to the use of a PD-type controller, as well as the physical characteristics of the electric motors and the mechanics of the robot itself. During this time, the motor passes through the moment of zero acceleration. Furthermore, it starts from zero acceleration, while lifting the robot’s body, i.e., it overcomes the weight of the structure. This delay can be eliminated if the two phases are planed with a time overlap.

**Figure 11:**
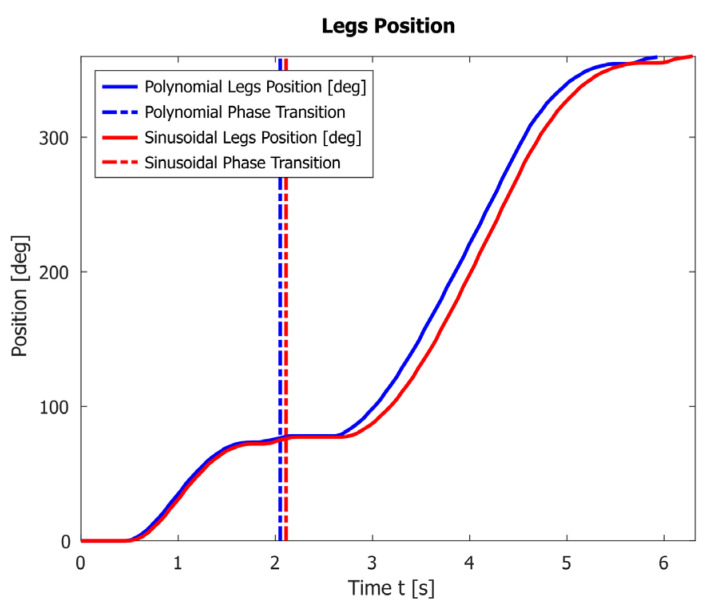
A comparison of the legs’ positions when using polynomial and sinusoidal law.

A comparison of the angle change φ (legs’ positions) when using polynomial and sinusoidal law is shown in Figure 11.

A video with some of the experiments is available at the following link: https://youtu.be/zo1276JLs0k (accessed on 20 January 2023).

## 5. Discussion

The robot “Big Foot” is an innovative design with minimal degrees of freedom and as such it is difficult to make a direct comparison with other designs. For example, ref. [32] deals with similar issues when trying to develop control algorithms for the joints of bipedal walking robots. They approach the problems in three steps: planning method, mathematical modeling (dynamics), and control algorithms. While we also have a dynamical model of the design [30], the simplicity and static stability allows for a purely kinematic approach (with adequate support from sensory input), with the only restrictions being motor loads and impact shocks. Note that in [32], the author tried to solve similar problems (minimizing impact shocks) using similar methods (a PD controller) with a key difference being that they include force control methods.

In article [33], the authors conduct a simulation of a walking robot with a similar analysis. They present angles and angular velocities with and without impact, and their effect on walking speed. They, however, do not consider accelerations and impact shocks.

The presented theoretical and experimental results are in good agreement but there are some differences. A difference is observed between the calculated rotation angles $\varphi_{B}$ and $\varphi_{s}$ for the two phases and the experimentally measured ones from the motor encoder. In reality, the dimensions have inaccuracies as there are slacks in the joints as well as elasticities, which lead to a deviation of the actual values for the angles for the two phases. This experiment is important for accurate determination of the coefficients in the control laws.

The theoretically calculated intervals for the two phases of motion provided in Table 2 differ from the experimentally obtained results presented in Figure 9 and Figure 10. This is due to an inaccurate determination of the actual coefficients and the fact that the proposed model does not take into account the dynamics of the process. However, since the velocities are low, the inaccuracies from the dynamics are insignificant. The experimentally obtained values for $T_{i}$ are bigger than the theoretical ones.

Graphs in Figure 6 show that both proposed laws provide a smooth increase in velocity and acceleration and satisfy the initial conditions. However, the polynomial law completes one period for $T=5.38\left[s\right]$, which is $0.32\left[s\right]$ faster than the sinusoidal law. This is also confirmed by the result given in Figure 11.

The experimental results given in Figure 7 and Figure 8 show that under a motion with a constant angular speed $\omega_{1}=118\left[{}^{\circ}/s\right]$ of the link 4, which is close to the maximum permissible one for the motor, the accelerometer reports very high acceleration fluctuations during the transition process. This leads to significant loads on the robot structure, which are also visible in the attached video. Decreasing the speed leads to a reduction in accelerations and shock loads, but at a constant angular velocity it significantly reduces the speed of the robot.

The experimental graphs in Figure 9 and Figure 10 show the deviation of the angular velocity from the theoretical one. These deviations are largest at the transition points between the phases and at the maxima of the functions. The polynomial law executes one period in $T=5.99\left[s\right]$, which is $0.33\left[s\right]$ faster than the sinusoidal law. This difference is very close to the theoretically obtained value. Therefore, the polynomial law can be used to make the robot move faster.

Figure 7, Figure 8, Figure 9 and Figure 10 also contain the accelerations normal to the walking surface (labeled Z-axis) read by the accelerometer. One could notice that the application of polynomial and sinusoidal control laws (Figure 9 and Figure 10) lead to much lower values than the accelerations obtained with the maximum angular velocity given in Figure 7. These accelerations are close to those obtained in Figure 8 at the average angular velocity. An important advantage of both laws is that they significantly shorten the execution time of each period while keeping low values of accelerations along the Z-axis. This corresponds to small dynamic loads.

Figure 9 and Figure 10 also show some disadvantages of applying the sinusoidal and polynomial control laws. In the transition between the two phases, there is a delay, which is a result of two things: the motor needs to overcome a significant torque at low speeds, which is difficult for the DC motor to do; the PD controller tries to ensure the correct motor angle, with close to zero angular velocity. When the angular velocity increases sufficiently, the PD controller tries to “catch up”, as is evident by the blue line in Figure 9 and Figure 10. This leads to another difference between experimental and theoretical results, located around the maximal values of the angular velocity. The controller is trying to compensate the difference between the real and expected velocities, which leads to overshoot when the expected velocity rapidly changes at the maximum. Note that the difference is more dire for shorter periods. The first issue could be solved by using more powerful motors, but this necessitates changes to the mechanical construction and electronics of the robot. Both issues can probably be solved by the implementation of a more sophisticated controller (for example a full PID controller). The delay can also be eliminated if the two phases are planed with a time overlap. Improvements in those directions are planned for future work.

## 6. Conclusions

We present a theoretical and experimental approach for the control of an innovative design of a walking robot with only two degrees of freedom, named “Big Foot”. Our approach aims to reduce shock loads while trying to maximize walking speed over a flat surface. The proposed algorithm utilizes a PD controller using the robot’s tactile sensors and encoders to determine the transition between the phases of walking, and the motor’s angular velocity. Three different laws of motion were compared: constant angular velocity, polynomial, and sinusoidal. Theoretically and experimentally, it is shown that the polynomial law leads to higher walk speed compared with the other laws, while maintaining low motor loads and low impact shocks.

The flaws in experimental realization could be eliminated by using a more complicated control algorithm (for example a full PID controller), more powerful motors, or more sophisticated laws of motion (time overlap between different phases of walking). The proposed scheme can be generalized in two ways: by considering collision and obstacle avoidance; and by walking in an uneven and/or unstructured environment. The proposed approach may be applicable to the control of the walking mechanisms of similar mobile robots.

## 7. Patents

Chavdarov I, Tanev T, and Pavlov V. Walking robot. Patent application № 111362. Published summary—Bulletin № 6, 30 June 2014, p. 11, in Bulgarian.

## Figures and Tables

**Table 1:** Processes during the different phases of motion.

Phase	Motionless Parts	Instantaneous Center of Velocity for Arm 4	Time Interval	Movement of the Robot	Active Sensor
I	Feet 5L and 5R	Point A (Figure 3)	$T_{1}=t_{1}-t_{0}$	Yes	Encoder
II	Base 1 and body 2	Point B (Figure 3)	$T_{2}=t_{2}-t_{1}$	No	Tactile sensors, encoder
Transitional process	Undefined	Undefined (instantaneously changes from A to B)	Undefined short time	Undefined	Accelerometer

**Table 2:** Minimal duration of phases I and II for the considered control laws.

Law	Minimal Duration of the Phase [s]		Period *T* [s]
	$T_{1min}\mathrm{for}\;\mathrm{Phase}\;I$	$T_{2min}\mathrm{for}\;\mathrm{Phase}\;\mathrm{II}$	
Polynomial	1.84	3.54	5.38
Sinusoidal	1.92	3.78	5.70

## Data Availability

Not applicable.

## Supplementary Materials

The following supporting information can be downloaded at: https://youtu.be/RYRJZcYdIX0—Video S1 (Figure 2); https://youtu.be/zo1276JLs0k—Video S2 (Figure 11).
